# Cell-Free DNA Fragments as Biomarkers of Islet β-Cell Death in Obesity and Type 2 Diabetes

**DOI:** 10.3390/ijms22042151

**Published:** 2021-02-21

**Authors:** Marilyn Arosemena, Farah A. Meah, Kieren J. Mather, Sarah A. Tersey, Raghavendra G. Mirmira

**Affiliations:** 1 Kovler Diabetes Center and the Department of Medicine, The University of Chicago, Chicago, IL 60637, USA; marilyn.arosemena@uchospitals.edu; 2 Department of Endocrinology, Edward Hines Junior VA Hospital, Hines, IL 60141, USA; farah.meah@va.gov; 3 Department of Medicine, Indiana University School of Medicine, Indianapolis, IN 46202, USA; kierenmather@gmail.com

**Keywords:** β-cell biomarkers, β-cell dysfunction, C-peptide, insulin, type 1 diabetes, type 2 diabetes

## Abstract

Type 2 diabetes (T2D) typically occurs in the setting of obesity and insulin resistance, where hyperglycemia is associated with decreased pancreatic β-cell mass and function. Loss of β-cell mass has variably been attributed to β-cell dedifferentiation and/or death. In recent years, it has been proposed that circulating epigenetically modified DNA fragments arising from β cells might be able to report on the potential occurrence of β-cell death in diabetes. Here, we review published literature of DNA-based β-cell death biomarkers that have been evaluated in human cohorts of islet transplantation, type 1 diabetes, and obesity and type 2 diabetes. In addition, we provide new data on the applicability of one of these biomarkers (cell free unmethylated *INS* DNA) in adult cohorts across a spectrum from obesity to T2D, in which no significant differences were observed, and compare these findings to those previously published in youth cohorts where differences were observed. Our analysis of the literature and our own data suggest that β-cell death may occur in subsets of individuals with obesity and T2D, however a more sensitive method or refined study designs are needed to provide better alignment of sampling with disease progression events.

## 1. Introduction

Type 2 diabetes (T2D) is associated with metabolic derangements such as obesity, hypertension, and accelerated atherosclerosis. A rise in the prevalence of diabetes specially in those under 30 years are an increasing source of concern [1]. T2D results from inadequate insulin production to meet peripheral insulin demands, however the underlying etiology of this dysfunction is multifactorial. There are several proposed mechanisms underlying the inadequacy of insulin production in T2D (reviewed in [2]): (1) β-cell dysfunction arising from cellular stress resulting in failure to produce and secrete adequate insulin, (2) β-cell death resulting in reduced cellular mass; and (3) β-cell dedifferentiation resulting in loss of β-cell identity and failure to secrete insulin. Whether these three mechanisms are independent of one another or are mutually exclusive remains an unresolved issue. For example, under conditions of stress such as increased insulin demand or inflammation in the context of insulin resistance, the capacity of the endoplasmic reticulum (ER) can be overwhelmed resulting initially in inadequate protein production and loss of function; more prolonged ER stress can lead to the activation of apoptotic pathways, resulting in cellular death [3,4]. Similarly, de-differentiation is thought to occur in T2D when β cells are exposed to increased glucose and/or fatty acid levels, leading to excessive oxidative stress and eventual loss of key transcriptional regulators that define β-cell identity [5]. 

Precisely if and when β-cell stress, de-differentiation, or death—events typically identified by tissue pathology—might occur in the course of T2D would be important to ascertain, as these events could be targets for therapeutic intervention. Yet, a major challenge in humans is the inaccessibility of pancreatic tissue from which to identify these events in a longitudinal, minimally invasive fashion. Biomarkers of β-cell stress that have been proposed include the measurement of proinsulin/C-peptide ratios or pro-islet amyloid polypeptide/islet amyloid polypeptide (pro-IAPP/IAPP) ratios in the circulation [6,7], which result from impaired or abnormal prohormone processing events. Moreover, the presence of high circulating proinsulin levels may also report loss of β-cell identity [8], although specific circulating markers of de-differentiation have yet to be identified. The utility of proinsulin and pro-IAPP as markers of β-cell stress in diabetes have been the focus of other recent reviews [9,10]; here, we will review the occurrence and measurement of β-cell death by cell-free circulating DNA in diabetes, with a focus on T2D, and their implications in disease pathophysiology. Other recent reviews offer synopses using other type of nucleic acids, such as RNA species, as biomarkers of β-cell death [9,11,12,13].

## 2. Islet β-Cell Death in Diabetes

The loss of β-cells is considered a key to the pathogenesis of both type 1 diabetes (T1D) and T2D. In the case of T1D, aggressive autoimmunity with influx of cytotoxic T cells into islets results in β-cell stress, deterioration of β-cell mass, and evidence of cellular apoptosis on tissue pathology [14,15]. Although β cells persist in even longstanding T1D [14,16,17], there has been little controversy in the literature whether that loss of β cells occurred by any other mechanism than death. By contrast, the mechanisms underlying β-cell loss in T2D are not entirely resolved. The loss of β-cell mass in T2D was demonstrated in the 1950s in elegant post-mortem studies of Ogilvie [18], and confirmed in subsequent more recent work [19,20]. Some studies have shown evidence of apoptotic β cells in cadaveric pancreas from subjects with T2D [19,20], and others suggest that loss of β-cell mass might arise from de-differentiation of β cells [21]. This de-differentiation is also thought to cause a change in function, with production of glucagon and/or somatostatin [21]. Mouse models suggest that both events, β-cell death and de-differentiation, may occur through the course of disease, but appear to be temporally distinct, with the former occurring early in disease pathogenesis and the latter occurring late [22]. The resolution of diabetes pathophysiology in human disease could have implications for β cell-targeted therapeutics and the timing of such interventions.

## 3. Development of DNA-Based Biomarkers of β-Cell Death 

There has been increasing interest in identifying circulating biomarkers of β-cell death, driven primarily by the notion that such biomarkers may identify cellular events in pre-symptomatic T1D that precede even the appearance of autoantibodies. With the focus on initiating therapeutic trials in the presymptomatic phases of T1D, the development of such biomarkers has been gaining traction in recent years. Based on approaches previously developed for cancers and identifying fetal DNA in maternal circulation, it was proposed that DNA species liberated from dying β cells could be measured to correlate the appearance of such DNA with concurrent β-cell death [23]. Initial studies focused on the gene encoding preproinsulin *(INS*), which is expressed almost exclusively in islet β cells. The insulin gene exhibits cell-specific methylation at cytosine bases located throughout the coding and non-coding regions, such that these cytosines (located at “CpG” sequences) are mostly methylated in non-β cell types but unmethylated in β cells [24]. Upon chemical bisulfite treatment, unmethylated cytosines at these CpG sites are converted to uracils, whereas methylated cytosines are unaffected; hence, the appearance of *INS* DNA fragments in the circulation wherein cytosines are unmethylated can be detected by sequence-discriminatory PCR techniques, thereby allowing attribution of this DNA to β cells. This concept has formed the basis for multiple assays that have been developed over the past decade (see Table 1 for a summary of these studies). These assays have spanned studies across mice and humans and have leveraged different CpG sites in *INS* and other genes, including *GCK, IAPP,* and *CHTOP*, as indicated in Table 1.

### 3.1. Application of β-Cell Death Biomarkers in Islet Cell Transplantation

The validity of differentially-methylated circulating DNA as biomarkers of islet β-cell death has been tested in islet transplantation settings in both animal models and humans, since it is expected that transplanted islets harbor a significant fraction of lysed β cells and that the immediate post-transplant period exhibits instant blood-mediated inflammatory reaction leading to islet death [25]. In immunocompetent mice, xenotransplantation of human islets results in the immediate release of unmethylated human *INS* DNA into the circulation, as measured by traditional quantitative PCR techniques [26]. Similarly, in humans, the levels of unmethylated *INS* DNA (or the ratio of unmethylated:methylated *INS*) increased acutely following islet transplantation, consistent with death of β cells in the immediate post-transplant period [27,28,29,30,31]. Notably, this signal subsides within hours to days following transplantation, reflecting the balance between the half-life of the circulating DNA (likely hours to minutes) and the rate of ongoing β-cell lysis in any given individual. A study of recipients undergoing total pancreatectomy with islet autotransplantation revealed that unmethylated *INS* DNA levels decreased 1–4 h after pancreatectomy (concordant with loss of islet mass), but increased in the immediate post-transplant period [27]. Persistent post-transplant elevation of *INS* DNA in this study predicted greater hyperglycemia at 90 days, suggesting that early and persistent β-cell death may reflect a greater likelihood to longer-term loss of graft function. 

### 3.2. Evidence of β-Cell Death in T1D and in Subjects at-Risk for T1D

The earliest study to examine the occurrence of β-cell death in T1D utilized a nested PCR-based approach to show that unmethylated *INS* (relative to methylated *INS*) was elevated in a small cohort of youth with T1D (N = 5, onset within 1 year) compared to age-matched healthy donors [23]. The earliest assays in humans that focused on the use of traditional quantitative PCR-based strategies [28,32] have given way to more recent studies that rely on digital PCR (dPCR) or on traditional PCR combined with direct sequencing [29,33,34,35] that provide both greater sensitivity and ability to estimate absolute circulating copy numbers. A recent study evaluated subjects undergoing total pancreatectomy and islet autotransplantation using three different assays that measured unmethylated *INS* DNA. The study showed that during conditions when high levels of unmethylated *INS* DNA were present, the technical reproducibility was good amongst the three assays (two with dPCR and one with sequencing) [31].

Using the more highly sensitive dPCR or sequencing-based assays, several studies have demonstrated elevated levels of unmethylated *INS* DNA in newly diagnosed T1D compared to control subjects [26,36]. Yet, only a few have examined evidence of β-cell death in subjects with either long-standing diabetes or in those at-risk for T1D. Using banked sera from subjects in the T1D Exchange registry, one study demonstrated elevations in both unmethylated *INS* and methylated *INS* in subjects from both C-peptide-positive and C-peptide-negative subjects with longstanding (≥9 years) T1D [37]. These findings seem indicative of the persistence of β cells in such subjects, where ongoing β-cell death may give rise to circulating unmethylated *INS* signals and ongoing systemic autoimmunity/inflammation may give rise to elevated methylated *INS* signals [38]. At least two studies have examined the occurrence of β-cell death in subjects at risk for T1D. One study, using samples from the TrialNet Pathway to Prevention cohort, showed that autoantibody-positive subjects who later progressed to T1D exhibited modestly higher average unmethylated *INS* compared to controls or those who did not progress to T1D [39].

A more recent study [38] examined autoantibody-negative first-degree relatives of those with T1D (a group that is at modestly higher risk of developing T1D compared to otherwise healthy controls) and demonstrated striking elevations of unmethylated *INS* in this group. Although the findings of these studies suggest that a pre-symptomatic period with active β-cell death may occur, these findings were not corroborated in another study that used a DNA sequencing approach to examine unmethylated *INS* in subjects at-risk for T1D [29]. Whether the discrepancy between these studies reflects underlying differences in the assays employed, the specific subjects tested, or features of when they were tested in an unknown disease course remains unclear. 

Apart from the *INS* gene, other genes have been interrogated for the ability to identify β-cell death in subjects with T1D. These include other genes that are known to be preferentially expressed in β cells (*IAPP* and *GCK*) [42,43] and another (*CHTOP*) identified from an unbiased screen [38]. In all these cases, elevations in the unmethylated DNA species were observed in subjects with T1D compared to controls, but the utility of *IAPP* and *GCK* genes, alone or in combination with other genes, in stratifying populations at-risk for T1D remains untested.

## 4. Applicability of Unmethylated INS across a Spectrum from Obesity to T2D

### 4.1. Assessment of β-Cell Death in Obesity, Impaired Glucose Tolerance (IGT), and T2D

Studies interrogating β-cell death across the spectrum from obesity to T2D in humans are more limited in number compared to those in T1D and, to date, have been restricted to cross-sectional studies. In one study examining overweight adult subjects with atypical ketosis-prone diabetes, only those with autoantibodies and evidence of β-cell function demonstrated elevations in unmethylated and methylated *INS* [44]. Arguably, the cohort represented by autoantibody-positivity and persistence of β-cell function are more akin to patients with early T1D, notwithstanding the presence of overweight. A more recent study by our group examined both methylated and unmethylated *INS* and *CHTOP* in obese youth across the spectrum of normal glucose tolerance to T2D; these categories included lean controls with normal glucose tolerance (NGT), overweight/obese with normal glucose tolerance (OB-NGT), overweight/obese with impaired glucose tolerance (IGT), and overweight/obese with T2D with and without evidence of autoantibodies [38]. Obese youth, as a group, showed statistically greater levels of unmethylated *CHTOP* and both unmethylated and methylated INS compared to lean control youth—findings suggestive of β-cell death in youth with obesity. When these subjects were further stratified into five subgroups (lean controls with normal glucose tolerance; overweight/obese with normal glucose tolerance; overweight/obese with impaired glucose tolerance; T2D-autoantibody negative; T2D autoantibody positive), no statistical differences in unmethylated *INS* or *CHTOP* were observed across these cross-sectional cohorts. This finding suggests that the overall increases in unmethylated *INS* and *CHTOP* in the obese youth cohort were not driven by any subgroup, but rather by other features shared by the obese group, such as insulin resistance, adiposity, or systemic inflammation. By contrast, the increase in methylated *INS*, also observed in cohorts with or at-risk for T1D [26,38], remains unresolved, though such increases have been proposed to reflect states of systemic inflammation, where turnover of non-islet cell types might account for the elevations of methylated DNA species [38].

### 4.2. Assessment of β-Cell Death in Adults with Obesity, IGT, and T2D

**Figure 1:**
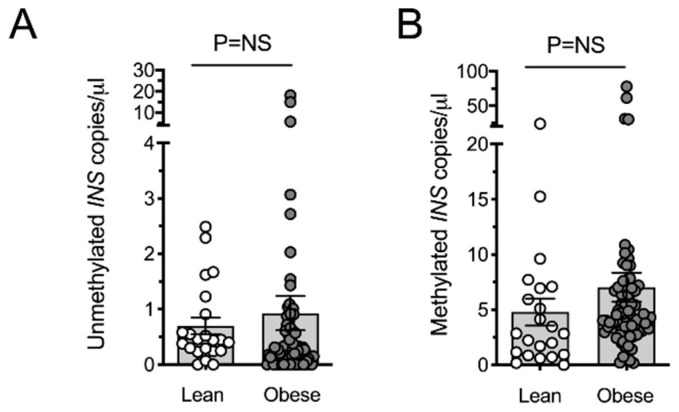
Circulating unmethylated and methylated *INS* DNA in lean and obese adults. DNA was isolated from serum of subjects and bisulfite converted as described previously [38]. Primers and dual-fluorescent probes for interrogating methylation at CpG position −69 at the human *INS* gene using droplet digital PCR were described previously [26,34]. Values were normalized for DNA recovery, and back-calculated to the volute of serum used in the DNA isolation (**A**) Circulating unmethylated *INS* DNA; (**B**) circulated methylated *INS* DNA. A Kruskal–Wallis (non-parametric) test was employed followed by a Dunnett’s post-test (to compare values to Lean controls). Statistical significance was assumed at *p* < 0.05. Data are presented as mean ± SEM.

**Figure 2:**
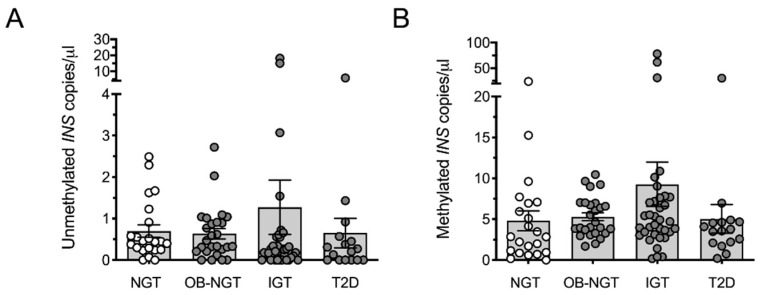
Circulating unmethylated and methylated *INS* DNA in lean adults with normal glucose tolerance (NGT) adults with obesity with normal glucose tolerance (OB-NGT), obesity with impaired glucose tolerance (IGT), and obesity with type 2 diabetes (T2D). (**A**) Circulating unmethylated *INS* DNA; (**B**) methylated *INS* DNA. A Kruskal-Wallis (non-parametric) test was employed followed by a Dunnett’s post-test (to compare values to NGT controls). Statistical significance was assumed at *p* < 0.05. Data are presented as mean ± SEM.

The preceding published studies are suggestive that obesity in youth may be associated with β-cell death. However, studies to date have not addressed its potential occurrence in adult cohorts. To follow up on this possibility, we describe here a new cross-sectional cohort of adults with obesity and/or T2D in whom we performed assays for methylated and unmethylated *INS* using banked serum samples. The assay we employed has been detailed in prior studies [26,34,38], and interrogated methylation at the CpG located at position −69 bp on the human *INS* gene relative to the transcriptional start site. Using a cross-sectional cohort of adult subjects (see characteristics in Table 2), we observed that obese individuals, unlike youth, did not exhibit statistically different levels of unmethylated or methylated *INS* compared to lean controls (Figure 1A,B). To assess if differences in adults can be observed based on glycemic control, we next stratified the cohorts by their levels of dysglycemia. Cohorts were stratified into 4 groups: Lean controls with normal glucose tolerance (NGT); overweight/obese with normal glucose tolerance (OB-NGT); impaired glucose tolerance (IGT); and T2D. As shown in Figure 2A,B, in this cross-sectional comparison, none of the groups showed statistically significant differences in unmethylated or methylated *INS* DNA compared to healthy controls, although some individuals in the obese group showed very high values. 

These data stand in contrast to those published by our group in youth [38], where obesity was associated with evidence of both β-cell death (unmethylated *INS*) and death or turnover of non-β cells, as evidenced by the elevations in methylated *INS*. Several possibilities might explain the differences between youth and adults. 1) The epigenetic landscape at the *INS* gene, or the rate of clearance of DNA fragments from circulation, might be altered by the aging process (or the de-differentiation of β cells) such that the assay itself is unable to pick up evidence of β-cell death in adults, 2) the prevalence and/or rate of β-cell death in adults might be below the detection limits of the assay, 3) they might reflect an accelerated inflammatory state across the spectrum from obesity to T2D in childhood, where stress and death of β cells may predominate in youth, and 4) it remains possible, as in mouse models [22], that β-cell death is an episodic phenomenon and that the cross-sectional nature of these studies miss the timing of cellular death. Because none of these possibilities can be categorically excluded, it continues to remain unclear if β-cell death is a prominent feature, particularly in adults with T2D. Despite the positive signals in youth, the occurrence of β-cell death may be a feature that reflects different endotypes of disease that remain to be categorized and at the very least requires confirmation from complementary assays or pathologic examination. Regardless, a major limitation in most studies of either T1D or T2D remains the lack of prospective sample collection, which would otherwise allow for temporal sampling and the ability to correlate β-cell death with specific outcomes in the populations.

## 5. Conclusions

Assays of β-cell death based on circulating differentially methylated DNA fragments have been gaining substantial interest in recent years. In the case of T1D, most assays have identified evidence of β-cell death in the early phases of the disease. It is notable that a recent study could not verify this occurrence using a sensitive PCR+sequencing approach [29], suggesting that either differences in assay sensitivity, the populations examined, or the CpG sites interrogated may be crucial variables to consider in the course of assay development. With respect to obesity and T2D, the utility of these DNA-based assays in identifying β-cell death remains unclear. The underlying pathophysiology of T2D is complex and protracted, with loss of both β-cell mass and function in the setting of systemic insulin resistance and inflammation. With recent studies now suggesting the possibility that β cells might be de-differentiating, it is possible that these de-differentiated β cells exhibit changes to the methylation status of their genes, thereby complicating detection of β-cell death. A key take-home message from this review of published studies to date is that DNA-based biomarkers have promise to detect β-cell death, but the assays used to measure circulating DNA fragments may still be limited in sensitivity and specificity in the context of T2D and obesity. As these assays are refined to enhance sensitivity and specificity, these DNA-based biomarkers may prove to be important tools that can help to distinguish the different endotypes of disease that define human diabetes.

## Figures and Tables

**Table 1:** Published studies utilizing differentially methylated DNA to interrogate islet β-cell death.

Study [Ref#]	Technique (Gene)	Subjects Studied (Number of Subjects)	Results
Akirav et al. [23]	qPCR (*INS*)	T1D subjects, onset within 1.5 yr (17), Healthy control subjects (11) Streptozotocin (STZ)-treated mice, NOD mice	-Assay can distinguish between islets and other organs - β-cell death in mouse models with increased β-death prior to a decline in insulin content -Increased β-cell death in T1D patients compared to controls
Husseiny et al. [40]	qPCR (*INS*)	No human subjects STZ-treated mice	-Assay can distinguish between β-cell DNA and other tissues -β-cell death before the rise in blood glucose levels
Lebastchi et al. [32]	qPCR (*INS*)	T1D subjects, onset within 0.6–6 yr (54), Healthy control subjects (19) T1D subjects treated with anti-CD3 monoclonal antibody or placebo (37)	-Increased β-cell death in T1D subjects compared to control subjects -Teplizumab treatment is associated with reduced level of β-cell death
Fisher et al. [41]	qPCR (*INS*)	No human subjects STZ-treated mice and NOD mice	-Assay can distinguish between β-cell DNA and other tissues -Increased β-cell death prior to the development of hyperglycemia and decreased β-cell death after increased hyperglycemia
Husseiny et al. [28]	qPCR (*INS*)	Islet transplantation subjects (6), Healthy control subjects (6) NOD mice	-Increased β-cell death starting at week 10 in NOD mice -Increased β-cell death on day 1 after islet transplantation
Usmani-Brown et al. [35]	dPCR (*INS*)	T1D subjects, onset within 1 yr (43), Relatives of T1D subjects with >2 aAb (26), Health control subjects (27)	-Improved specificity using droplet dPCR -Increased β-cell death in T1D and at-risk subjects compared to controls
Herold et al. [39]	dPCR (*INS*)	At risk subjects (20): T1D progressors (10), Non-progressors (10), High risk subjects (30): Diabetic OGTT (10), Dysglycemia (10), Normal OGTT (10), Islet autotransplantation (4), Control subjects (32)	-β-cell death was associated with decreases in insulin secretion -Detected increased β-cell death in at-risk subjects who progressed to T1D.
Fisher et al. [26]	dPCR (*INS*)	T1D subjects, onset within 48h (32), Obese subjects without diabetes (21) T2D subjects (17), Pediatric control (27), Lean adult control (15) STZ-treated mice, NOD mice	-β-cell death at T1D onset -No β-cell death detected in T2D or autoimmune disease
Lehmann-Werman et al. [36]	DNA Sequencing (*INS*)	T1D subjects, onset within 1–4 months (11), Islet-graft recipient subjects (10), Pancreatic cancer (42) and pancreatitis (10), Healthy control subjects (31)	-β-cell death at onset of T1D controls
Olsen et al. [42]	qPCR (*IAPP*)	T1D subjects (15), Healthy control subjects (11) NOD mice	-Detected β-cell death in recent onset T1D subjects
Tersey et al. [34]	dPCR (*INS*)	T1D subjects, onset within 48h (3), Healthy control subjects (3)	-Detected β-cell death in new-onset T1D
Bellin et al. [27]	dPCR (*INS*)	Total pancreatectomy with islet autotransplantation (25), Healthy control subjects (49)	-Decreased β-cell death after pancreatectomy -Increased β-cell death hours after transplantation -Persistent β-cell death predicted greater hyperglycemia at 90 days
Sklenarova et al. [43]	dPCR (*GCK* and *INS*)	T1D subjects, onset within 4 weeks (25), At risk individuals (14), Healthy control subjects (20)	-Unmethylated *GCK* DNA was more islet specific than unmethylated *INS* DNA -Increased β-cell death in autoantibody positive relatives compared with T1D and controls
Tersey at al. [22]	dPCR (*INS*)	No human subjects High and low fat diet-treated mice; STZ-treated mice	-Episodic levels of β-cell death during obesity
Mulukutla et al. [44]	dPCR (*INS*)	Ketosis prone diabetes (KPD) subjects (112)	-Increased β-cell death in patients with A+ β+ KPD compared to other KPD subtypes -A+ β+ KPD has a slowly progressive β-cell destruction
Neyman et al. [37]	dPCR (*INS*)	T1D subjects, long standing (90), Healthy control subjects (54)	-Increased β-cell death among both C-peptide (−) and C-peptide (+) subjects with longstanding T1D
Simmons et al. [45]	dPCR (*INS*)	At risk subjects (57), Control subjects (165)	-Autoantibodies were associated with increased β-cell death near diabetes onset -Increased β-cell death over time was associated with a younger age of T1D onset
Speake et al. [31]	dPCR, DNA Sequencing (*INS*)	Total pancreatectomy and islet autotransplantation (13), Control subject (1)	-Levels of β-cell death were correlated across different assays
Neiman et al. [29]	PCR-DNA Sequencing (*INS*)	T1D subjects (130), At-risk subjects (32), Healthy control subjects (218)	-No detection of β-cell death in autoantibody-positive subjects at risk for T1D, recent-onset type 1 diabetes or those with long-standing disease
Syed et al. [38]	dPCR (*INS* and *CHTOP*)	T1D subjects, onset within 48 h (43), At risk subjects (23), T2D subjects (56), Healthy control subjects (10)	-Increased β-cell death in youth with new-onset T1D and healthy autoantibody-negative youth who have first-degree relatives with T1D -Increased β-cell death in youth obese youth compared to lean controls

**Table 2:** Subject characteristics. Subjects are divided into categories based on oral glucose tolerance test (OGTT) 2hr-glucose concentrations: normal glucose tolerance (NGT, <140 mg/dL), obese-normal glucose tolerance (OB-NGT, <140 mg/dL), impaired glucose tolerance (IGT; 140–199 mg/dL), and T2D (≥200 mg/dL). Exclusion criteria: Metformin use four weeks previous, thiazolidinedione use six months previous, T1D, other diabetes, pregnancy, weight fluctuation six months previous, current or past tobacco use, acute or chronic illness, pulmonary disease, or use of antidepressants. Participants were provided written informed consent for screening and study participation. The study was approved by the Indiana University School of Medicine Institutional Review Board. OGTT: Oral glucose tolerance test.

	NGT	OB-NGT	IGT	T2D	*p* Value
Number (% male)	24 (54)	39 (62)	38 (58)	16 (68)	
Age (years)	40 ± 4.1	46 ± 1.9	46 ± 1.8	49 ± 2.8	0.30
BMI (kg/m^2^)	21.7 ± 0.57	33.8 ± 2.54	31.5 ± 1.32	38.7 ± 3.73	<0.001
Fasting glucose (mg/dL)	84.2 ± 1.30	90 ± 6	104 ± 10.5	136 ± 16	<0.001
2 hr OGTT glucose (mg/dL)	94.9 ± 8.04	104 ± 19	140 ± 24.5	262 ± 33	<0.001

## Abbreviations

dPCR	digital PCR
IGT	impaired glucose tolerance
NGT	normal glucose tolerance
NOD	non-obese diabetic
NOD-SCID	non-obese diabetic-severe combined immunodeficiency
OB-NGT	obese normal glucose tolerance
OGTT	oral glucose tolerance test
T1D	Type 1 Diabetes
T2D	Type 1 Diabetes
